# The reticular hypodermic venous system, the true integrator of the superficial venous system

**DOI:** 10.1080/07853890.2021.1897456

**Published:** 2021-09-28

**Authors:** J. Ovelar, J. Cédola, J. Merino

**Affiliations:** CIMED FUNDAMI Digital Anatomy Laboratory, La Plata, Buenos Aires, Argentina

## Abstract

**Introduction:**

An anatomical observational, retrospective and cross-sectional study of the Venotomographies performed at CIMED was carried out from January 2014 to the present, with 105 studies of patients of both sexes. The study methodology was multislice computed venotomography, which allowed to achieve a live anatomical study in several observational planes and making virtual dissections. The aim is demonstrating by means of virtual anatomy how the reticular hypodermic venous system (RHVS) connects the main superficial venous trunks to each other, to the perforating venous system and through them to the deep venous system.

**Materials and methods:**

The scanner used was a 64-detector Phillips multislice tomograph and the program chosen was Phillips' IntelliSpace Portal. To determine the characteristics of RHVS, it was divided the studied population into three groups: 1- without venous pathology, 2- with venous hypertension due to obstruction or compression, 3- in varicose recurrence.

**Results:**

The procedure used allowed to demonstrate that the RHVS produces a true integration of the entire superficial venous system, perforating venous system and through this to the deep venous system as well. **Discussion and conclusions:** The MCVT has proven to be a versatile and effective method of observing the entire superficial venous system in detail. The RHVS integrates both truncal venous systems, saphenous vein magna, saphenous vein parva and its main tributaries. It integrates all perforating veins with the reticular venous system. And it links the superficial venous system and the perforator with the deep venous system. This conclude affirming that the entire superficial venous system is a single anatomical unit integrated by the RHVS.
